# Notices of the Lunatic Asylums of Paris

**Published:** 1845-04

**Authors:** John Conolly

**Affiliations:** Fellow of the Royal College of Physicians, Physician to the County Lunatic Asylum at Hanwell


### NOTICES OF THE LUNATIC ASYLUMS OF PARIS, &c.

*In a Letter addressed to* JOHN FORBES, M.D. F.R.S.

PART II.

By JOHN CONOLLY, M.D.

**Fellow of the Royal College of Physicians, Physician to the County Lunatic Asylum at Hanwell.**

### CHARENTON.

The French physicians begin their labours at an early hour; and at eight o'clock on a fine morning in November I had the pleasure of accompanying M. Foville to the celebrated asylum of Charenton, situated a few miles east of Paris, on the elevated bank of the Marne. Sixteen years before, I had visited this asylum, of which Esquirol was then the chief physician. He succeeded the eminent Royer-Collard. The appointment was offered to Pinel in 1797, when Charenton was opened as an asylum for the insane; but he could not make up his mind, Esquirol says, to leave his poor patients and his pupils at the Salpêtrière. M. Royer-Collard was succeeded by Esquirol, at whose death M. Foville was appointed. Thus to revisit the same place, after even the short interval of a few years, is to become more sensible of the gradual changes which time is always effecting; of the removal of individuals, and the onward progress of all those works which successions of individuals are ordained to accomplish. Esquirol has been removed from the scene of his great and useful exertions; but the asylum to which fourteen of his latest and most experienced years were devoted is undergoing that entire renewal for which he so anxiously wished. Old and inconvenient structures are disappearing, and new and elegant buildings are in progress which almost assume the character of grandeur. Whilst these alterations afford opportunities for numerous improvements, both in the construction of the asylum and the management of the patients, strikingly conspicuous at present, when a part of the old building still remains, it is questionable whether so great an expenditure as is now incurred should have been applied to a building in such a site; the surrounding ground, although the asylum is on an agreeable eminence, being rocky, precipitous, and unfavorable to the formation of airing-courts, or obtaining space and productive soil for gardens affording recreation, or fields furnishing employment. These natural inconveniences are alluded to in M. Esquirol's description of the new part of the asylum on the female side, completed in his time, and which have still very great advantages over the older parts of the building. The attempt to adapt the buildings of old religious houses, fortresses, and prisons, to the purposes of an asylum is usually unsuccessful. In the case of Charenton, formerly a religious house, this was from the first detrimental to the old plan, and an adherence to the same locality seems to be almost equally so to the new.

The disposition of the older parts of the asylum of Charenton is so extremely irregular as to defy intelligible verbal description; that of the new, but yet unfinished part, appeared to have the general character of several detached quadrangles or courts, built on each side of a central church of Greek architecture. The material is a beautiful stone. On three sides of each of these courts or quadrangles are handsome buildings of two floors, and on the third side, to the south, a lofty iron railing, through which is obtained a fine and extensive prospect. This noble institution is chiefly destined to the reception of patients of the educated classes of society, or of the middle classes; literary and scientific men, artists, merchants, ecclesiastics, officers of the army and navy, persons in public offices, or their wives or children, when affected with insanity. They are received at different rates of payment, principally in three classes; and some, nominated by the Minister of the Interior, are admitted gratuitously. The annual payments range from 720 to 1300 francs per annum. Some patients are placed in the asylum at reduced rates of payment, also on the recommendation of the Minister of the Interior, and pay only one half or one third of the usual charge. For these an annual sum is paid out of a public fund, by the government. Fourteen beds are reserved for the pauper lunatics of the canton. At present the number of patients is about 450; but with the new buildings a considerable enlargement is contemplated, a circumstance certainly to be regretted. The general adaptation of the building is suitable to those intended to inhabit it; but although its character, taken altogether, and its fine situation are striking, the appearance of the square courts affects the spectator unfavorably; they are extremely clean and command fine prospects, but are not sufficiently spacious entirely to remove the idea of a prison, derived from the elevated and apparently inaccessible situation of the asylum. I found such to be the impression made on the Parisians, and it is, perhaps, disadvantageous to the institution. All the interior arrangements are, however, most unobjectionable. The single rooms for patients, of which there are many, are of good size, excellent height, have large cheerful windows, and are comfortably furnished. The number of large dormitories appeared to me to be very small in proportion to the single rooms, at least in the new building; and I think M. Foville scarcely participates in the general partiality for them which prevails in France. In different parts of the asylum, the furniture and arrangements of the rooms are of course various; but there is great comfort throughout all those of recent construction. The apartments for several of the patients have a small room attached to them for an attendant; and I was glad to see that these were neither dark nor inconvenient, as is too generally the case in the older asylums, in which the attendants received no consideration. The truth slowly makes its way that good attendants make good patients; and that the way to make good attendants is to choose respectable persons and to make them comfortable. The importance of this very simple truth in asylums is immense. In all the French asylums, the beds are worthy of admiration. At Charenton almost all the bedsteads are of iron. The diet, varied according to the habits of the patients, is abundant and excellent. Patients of a certain rank dine with the director, or in their own apartments. All the patients are neatly and becomingly dressed in clothes provided by their friends. The military and naval patients receive clothing from the establishment. Great order and exactness characterizes all the arrangements relative to these matters; but they are too many for detail: although, like everything in this well-governed institution, deserving of the utmost attention from the directors of similar establishments.

As many or most of the female patients at Charenton are gentlewomen, and many of the men are old officers, or persons accustomed to the various impressions of social life in classes enjoying a certain degree of comfort, a great variety of resources becomes not only available in their treatment but necessary. Among the women, all the kinds of needlework form a large part of their daily occupation; but reading, and drawing, and music contribute to diversify their time. Gardens and terraces of considerable variety and beauty offer them inducements to take exercise out-of-doors. Among the men, reading and writing are the most prevalent modes of employing themselves; music and billiards are among their amusements. Many, as in all asylums, prefer walking about to any occupation. Some of the most tranquil women were in large and comfortable day-rooms, in which there were several tables and chairs; but they seem, as usual with the insane, to lead an isolated life; many living much in their rooms, and few appearing to associate intimately with the rest. Most of the men seemed to live much alone, and the apartments of some had the air of a quiet study or bureau, in which our visit created a kind of temporary interruption. Patients of the higher classes fall most easily into a monotonous course of life when insane; and the most active ingenuity of a director can scarcely redeem them from it. In one room, used as a school-room, a patient was acting as schoolmaster or lecturer to the rest; demonstrating the situation of various countries on a large map, which he did quite correctly, although with occasional hesitation: and it was interesting to notice his successful struggles now and then to deliver himself from some difficulty of expression, or some temporary forgetfulness, or some transient confusion in his thoughts.

Every evening some of the patients, named by the physician, and usually including the convalescents, are assembled in a kind of common room, or *salon*, for the sake of society and social enjoyments; some of the officers of the asylum being always present. Something of this kind is much wanting in almost all our large English asylums. The opportunities and advantages of such recreation are too rare; for their effects when enjoyed are very remarkable and evidently salutary. Constant work and formal discipline, transferred from prisons and workhouses, cast a gloom over many patients and create much avoidable discontent. It seems to be forgotten that most of the minds to be dealt with in asylums are *spoiled* for continuous application, efficient labour, or invariable order and propriety; and scarcely less forgotten that many require to be roused into a capacity for exertion rather than to be stimulated to exert themselves regularly. Many young patients, especially, are to be noticed in the wards of asylums, of whom the history is, that after a short attack of maniacal excitement, they have become listless, apathetic, and indolent; but these patients understand everything that is said to them, and many of them are neither affected with delusions or bewilderment of mind. The brain seems to have lost much of its energy; and their tendency is towards total imbecility. Such patients are unwilling, generally indeed unable to work; and therefore they fall into disfavour with all but medical observers. Under an austere system they perish; falling gradually into fatuity or dementia. They require encouragement; and for them cheerful recreation, frequent amusements, various exercise, mingled often with kind remonstrances, are remedial. They are more benefited by drawing, music, and children's games, than by medicine. Among patients of a higher class, as at Charenton, social appliances become of wider applicability. In all these attempts, however, as in the whole treatment of insanity, a moderation must ever be preserved. Lunatics bear no violent impressions with benefit, or even with impunity. The appliances should be very frequent; but never long continued, and never excessively exciting.

M. Foville's writings on Insanity and on the Nervous System are so well known in England, that it is scarcely necessary to say that he is a physician of great powers of observation and reflection, anxious for every improvement, but careful of hasty innovation, and habitually exercising an accurate judgment on whatever falls within the department of medicine in which he is now so experienced. The annual Reports of Charenton, presented to the Minister of the Interior, are not published; a circumstance which probably deprives the public of some of those fresh and valuable impressions suggested in the constant attendance of a physician in an institution presenting a large and interesting field for observation; and the value of which might be gathered even from the casual remarks made by this accomplished physician in the course of the long morning visit in which I was permitted to accompany him. His general treatment of his patients appeared to me to be essentially the same as that adopted by English practitioners in mental disorders; consisting of means of regulating the functions of the digestive organs and the skin, equalizing the circulation, allaying nervous disturbance, and soliciting a renewal of natural cerebral exercise. In his intercourse with his patients he is extremely mild, composed, and judicious; never attempting too much at once, or endeavouring to force powers that require considerate direction rather than stimulus; and evidently doing only what he considers to be really useful, and nothing from ostentation. Thus M. Foville does not assume the office of lecturer as he goes his rounds, or make the least attempt to convince the visitor that he is enjoying a great privilege. But the whole demeanour of a sensible physician when he goes through the wards of an asylum is instructive; everything has its meaning and its object. Vivacious visitors too often forget this; talk too much, suggest too hastily, discuss too earnestly, and observe too little; oblivious that the physician is actually applying various proportions of mental remedy at every step. I need scarcely say that in my capacity as a visitor I was careful not to err in this way.

It appeared to me that M. Foville was particularly observant of the general character, temperament, and configuration of his patients, and, although a very guarded phrenologist, had paid more than common attention to the relations of the skull to the development of the principal divisions of the brain. His anatomical researches and views respecting the physiology and pathology of the cerebro-spinal system have lately been given to the public in a valuable Treatise, reviewed in your Twenty-fifth Number, and of which it is unnecessary for me to speak further. M. Foville has made curious and, I believe, original observations on the shape of the ear in different forms of insanity, and has noticed an analogy or resemblance between the development of different portions of this organ and the brain of the patient. Of these views he was so obliging as to give me some explanation, illustrated by an extemporaneous diagram, and afterwards by corroborative examples. In some of the cases of dementia, or of the lowest degree of intelligence, the flatness and defective form of the helix, anti-helix, and tragus, and the disproportionate enlargement and pendulosity of the lobe of the ear, and rounded clumsy shape of the outer edge of the auricle, were very striking. Subsequent observations have led me to believe these views to be exact as well as curious; and they exemplify the abundance of external evidence available to the physician in relation to internal disorder. The diagnostics of Lavater fell into discredit by their idle and hasty application in the hands of his followers; but there are remarks in that great observer's works, even on so minute a matter as the natural growth and disposition of the *hair*, of the value of which I have only become conscious since my own observations have been accidentally directed to the hair as one of the external characteristics of melancholia, mania, and imbecility. Those familiar with the patients of any asylum can scarcely have failed to observe how frequently the disposition of the hair during the whole of a long maniacal paroxysm receives a peculiar modification; becomes raised, bristled up, *herissé*, and disordered beyond the control of comb or brush. The heavy masses of smooth hair in the melancholic must also have attracted attention. These characteristics are well portrayed, in some of the plates attached to Esquirol's work; and also in a spirited engraving entitled 'Narrenhaus.' It is not necessary to magnify the importance of these things; but they are not to be despised. They long to the studies of which Celsus says, *adjuvant excitando artificis ingenium.* Not very long ago, M. Foville was called upon by an intelligent and philanthropic person, who appeared to take much interest in the management of lunatic asylums; and he was greatly struck with a conformation of ears in this gentleman which he had never previously observed, except in cases of mental irregularity or disorder. I happen myself to know that the individual who was the subject of this observation has had several attacks of insanity; and although now at large, and exhibiting considerable mental activity, has repeatedly been in confinement; circumstances of which M. Foville had no knowledge when he remarked what seemed to him to be an anomalous peculiarity.

I did not see at Charenton any cases of an actual disease of the external ear which seems to be especially if not exclusively allied with cerebral disorder, being perhaps only observed in the insane. It consists of general but not acute inflammation, and slow enlargement, by thickening and effusion, of the whole of the outward ear; sometimes attended with much suffering; often becoming mitigated and then renewed, and never wholly removed, but not affecting the sense of hearing, except by mechanical obstruction of the meatus. There are always some examples of this affection at Hanwell, chiefly among the male subjects; but it cannot be clearly associated with any one original form of disease of the brain. It is allied in one case with epilepsy and weakness of the mental faculties; in others with general paralysis, and the incoherence consequent on mania; in others with confirmed dementia.

At Charenton, as in all large asylums, there are many examples of the form of paralysis, first fully described by M. Calmeil, and now generally known to the physicians of such institutions, although little recognized out of them, and called paralysie générale. M. Foville does not appear to be at all more sanguine than myself with respect to the means of cure of this form of malady. Daily experience confirms my opinion that it is invariably connected with irremediable organic change in the membranes or substance of the brain, or with effusions too extensive to be removed.

There was more agitation in the wards devoted to the least tranquil of the female patients at Charenton than I had observed at the Salpêtrière; but no great violence of conduct or manner: one apartment only presented an unfavorable exception, an apartment belonging to the old portion of the buildings allotted to the most turbulent. In this room I was strongly reminded of what was once familiar to me at Hanwell. The room seemed on entering it to be filled with noisy voices. Four or five of the female patients were sitting in those heavy coercion-chairs which are now rarely to be met with; one was leaning back in the chair with an air of sullen depression, and quite silent, appearing, indeed, to be in a state of mere dementia. Two or three others were struggling and declaiming with great vehemence and loudness; and another was talking very volubly but with perfect good humour. I was in some degree prepared for this spectacle by having just read in the 'Quarterly Review' that M. Foville was opposed to the non-restraint system; a subject on which I had not heard his opinion. But still it appeared to me almost as shocking as if I had never witnessed such a scene before, and had forgotten the dangerous ferocity of the daily occupants of the old coercion-chairs at Hanwell, and the wild careering of patients bound up in strait-waistcoats, and the shouts, oaths, threats, and curses which used to greet us every morning in the refractory wards, until the abolition of violent methods of repression caused such scenes to be unknown, and any approach to them rare and of short duration. As M. Foville did not make a single remark on these cases, or on the subject of restraints, I felt it incumbent upon me to maintain an equal reserve. I recollected that when he visited Hanwell in 1840, the great experiment of dispensing with restraints there, although previously made at Lincoln, was only in the first stages of its progress, and embarrassed by great opposition and difficulty. I thought it probable that he might have read the recent Report of the Commissioners in Lunacy, in which circumstances were alluded to, calculated without explanation to throw discredit on the non-restraint system, as practised even now at Hanwell. Otherwise, to a physician of so much good sense, and apparently so unprejudiced, I think I could have shown that no inconveniences were avoided by the restraints imposed on these poor women, which could not have been better obviated by less objectionable means; whilst some inconveniences were incurred by the use of restraints, that might have been advantageously and altogether avoided.

It appeared, from such inquiries as I had the opportunity of making, that in each of these examples of the use of the restraint-chair, the reason assigned was precisely similar to what is always alleged where restraints of any kind are still habitually employed. One patient would tear her clothes if at liberty; one would roll about the floor; one would break the windows; one would undress herself. Such reasons may surely be pronounced to be most unsatisfactory; each of these habits admitting of no very difficult remedy without restraints; and the tumult, the discontent, the air of neglect, the bad smells, prevailing in this as in every room where restraints are often used, constituting evils far greater than those which it is employed to prevent. Almost when writing these observations I have before me a fine old man, formerly a soldier, and recently brought to Hanwell, as too many are brought, merely to die. Broken down in constitution by service in a bad climate, he became enfeebled in body and mind; but, as his poor wife informs us, he was cleanly in his babits, and able to move about until lately fastened down in bed for weeks in one of the houses in which restraints are yet used with impunity; since which time he has become unable to walk, is suffering from ulceration of the back, is unable to attend to cleanliness, and lies helpless on a bed from which not all our care will ever make him rise again. I naturally mention this case because I have just been grieved by the sight of it; but similar instances are continually presented to our attention.

As M. Battelle has been furnished, at his express request, with patterns and models of all our Hanwell devices for meeting every inconvenience without tying the arms and legs of our patients or shutting them up in chairs, I hope he will be able to convince the officers of the asylums over which his administration extends, of their efficacy as well as their simplicity; and that their own experience will soon satisfy them of their preferableness to violence in any form. They cannot but find, that if a patient tears her clothes, it is not impracticable to dress her in strong clothing, not easily torn. If bedding and blankets are destroyed, strong cases will protect them; which restraints do not. If a patient undresses herself, the substitution of a small lock for buttons or strings, securing the dress at the neck or waist, and at the wrists, is readily accomplished and renders undressing impossible. Dresses of a peculiar form easily obviate the removal of blisters, dressings, &c., in cases requiring them, and remedy many inconvenient or uncleanly habits. If some patients take delight in breaking windows, it is easy to guard such windows as are exposed to these accidents by wire-blinds of a light construction, between which and the windows plants may also be placed, and kept from damage. If they feel a wild gratification in rolling on the floor, it is easy to have a soft covering for the floor. If, more desperate, they would dash themselves against the walls, which they very rarely indeed think of doing, it is not difficult to pad and cushion the walls of their rooms. If they will not lie down, or if from epilepsy or weakness they fall out of bed, it is very easy to make the whole floor a bed, from which they cannot fall, or on which they can walk, without danger, until they prefer lying down. For all cases of extreme violence, seclusion, or free exercise with proper superintendence, are the only useful measures not strictly medical; if they fail to produce tranquillity, violent restraints will render it still more unattainable. There is no difficulty and no danger which may not be met by analogous contrivances, which have now been successfully used in several English asylums for some years. And the general results of these contrivances are, that the patients become calmer, more apt to receive the impressions of all humane treatment; that the attendants do not become nursed in and inured to inhumanity; and that the *health* of the patients, and their cure, become the first considerations. They enjoy the free exercise of their limbs; and yet, in the end, fewer windows are broken; fewer violences of all kinds are committed ; the fierce excitement of the wards disappear; the life of the lunatic is rendered more tolerable, and that of the attendants and officers less anxious.

All my remarks have sufficiently shown that I think the general treatment of the patients in the French asylums is kind and judicious; but on recalling what I witnessed, I feel impressed with an opinion that the practice of resorting to restraint is not without its usual influence on the physicians who do so, although they may be somewhat unconscious of it. The practice tends to deaden the feelings to the actual condition of the patient,—an afflicted, suffering person; weak, wayward, and indolent, perhaps malicious or vicious, and yet innocent; above all, helpless and unprotected, except by the physician and those whom he employs. In illustration of this, I will merely mention that the use of baths of all kinds, but especially in their severest forms, is professedly resorted to as much and as often for a punishment as for a remedy. That the shower-bath and the douche have a moral, as well as a physical effect on an insane patient is undeniable; and that both are, in certain cases and with certain precautions, useful, with both these intentions, may also be granted; but it is no less certain, that to resort to either of them solely as a moral remedy, or as a means of repression, is a practice fraught with peril to the humanity of the practitioner, and liable to cruel abuse in the hands of his attendants. So used, it may become an instrument of torture, not a remedy; and instead of being employed to cure irritation of the brain may be applied to subdue and alarm the obstinate or the frantic, and even to force a poor creature, whose energies are oppressed, to undertake work only adapted to a healthy man. If the principle is once admitted that punishment and mortification are among the proper remedies for the insane, and that they ought to work, whether they work willingly or not, the austerity of cold and unfeeling officers will carry it to detestable excess. Combining it with the economy so important to all governing bodies which desire to conciliate the public, it becomes a pretext for lowering the diet, for neglecting the clothing, for withholding needful extras, except as the price of labour. For such offices, the effect of which is concealed, the officers of an institution may easily gain favour when really least deserving it. Certain it is, that the use of restraint seems, by some fatality, to be associated with innumerable negligences, of all which the patients are the victims. But much cold-hearted negligence, much severity even, may long and successfully prevail where restraints are ashamed to show themselves.

I must not, however, be unjust to Charenton, or to the asylums of Paris. When I recollect that in the Salpêtrière and the Bicêtre there are no resident physicians, the progress already made in abolishing restraints excites my surprise. Knowing the difficulties of the task, even to those constantly on the spot, I cannot sufficiently admire the courage, as well as the humanity of those who make the attempt in asylums in which they can only spend a few hours in a week, and to which the resources of their minds, and the suggestions of their kindness, can consequently have but transient application, and with faint results. In all the English and Scotch asylums in which restraint has been wholly abolished, there has been a resident medical officer zealous for its abolition; ready at any hour to make a prompt investigation into all the supposed obstacles to its success, and enabled to counteract opposing and sinister influences. I do not think its abolition has been effected, on a large scale, in any other circumstances.

These reflections are connected with the very important subject of the government of asylums, concerning which the most defective notions seem to me to prevail. In England no two asylums are governed on the same principle; aud the rules of many are constantly changing. In Ireland, although the officers have the advantage of acting under fixed regulations, which cannot be capriciously and suddenly changed, the government of asylums is intrusted to non-medical persons, not always persons of education. In our English county asylums, the qualification for being elected a governor is the accidental one of being in the commission of the peace. In the great charities for the insane, the office of governor is purchased. These arrangements seem scarcely adapted to the management of institutions differing from most others, in the necessity that exists for everything being done with an invariable regard to the state of mental and bodily infirmity with which every individual admitted into an asylum is more or less affected. Nothing is more certain than that from the laying of the first stone of an asylum to the completion of the rules for all who are to have authority in it, whatever is done without regard to this speciality will be done wrong.

There are many things in the constitution of Charenton deserving of attention; particularly at a time when there seems to be a prospect of every county being provided with a public asylum. There is a director, to whom all the economy of the asylum is subjected; he is appointed by the Minister of the Interior, and has himself the appointment of the treasurer, steward, architect, &c. There is a superintending committee, also named by the minister; they make visits of inspection, and report on what seems to them to require attention. The director carries all into effect. The chief physician and chief surgeon are also appointed by the minister of the interior. An assistant physician, and a medical officer called an inspector of health, are appointed by the physician, and also a limited number of house-pupils, each admitted for three years, who assist in all the medical duties. The director in no way interferes with the physician's department; they consult together on all general matters; but the physician entirely regulates the classification of the patients, their treatment, general and particular, and directs the mode of employment suitable to each. The physician is accompanied in his visits by one of the other medical officers and one or two of the house-pupils: they are accountable for all his directions and prescriptions being attended to, and they keep the journals and registers of the asylum.

All these arrangements seem well designed to ensure the appointment of competent officers; and to secure a just attention to the economic and general details, without producing a hurtful interference on the part of the director with the important functions of the physician, who has the entire control over everything comprehended in the medical, physical, and moral regimen of his patients; is the real head of the medical staff, and solely regulates the placing of the patients on their arrival and afterward. He prescribes the kind of employment and the degree of liberty, exercise, &c. proper for the patients, and is the only acknowledged judge of their fitness to be recommended to the religious care of the chaplain. The regulation which permits a certain number of young men to live in the asylum for a time as house-pupils is not only the means of training up useful officers for asylums, but enables the chief physician to keep fuller and more accurate records of the cases, and to preserve many observations of value which no one else has time to register with regularity. This admirable regulation prevails at Gloucester and at Glasgow, but not in the larger asylums of Bethlem, St. Luke's, Middlesex, or Lancaster. The chief physician at Charenton has also the chief control of the attendants, both male and female. No officer divides the power with him, or counter-orders his directions. Each female ward has a head attendant, by whom all the duties usually assigned in England to the matron on the female side, and for which no special officer is ever deemed necessary on the male side, appear to be very efficiently performed. Nothing, in short, that can affect the condition of the patients is done without being first submitted to the physician. That some womanly duties of kindness must be pretermitted by the absence of a matron is without doubt to be feared; but I can imagine reasons of great weight for the determination come to in the French, and recently in some of the American asylums, for having neither male nor female officer who is encouraged to act without medical control.

It is probable that for the salutary power now given to the physician at Charenton, he is in some degree indebted to the annals of the asylum, when, at a former period, a director was not in such friendly relation with the physician as the present experienced and liberal director, M. Palluy, under whom the asylum is undergoing its present renovation, appears to be with M. Foville. In M. Esquirol's account of Charenton there is mention made of a period when the director of the time utterly disregarded the opinions and authority of the physician. Difficulties of all kinds were thrown in the way of medical investigations. Statistics in every shape were discouraged or forbidden. The director, aided by two assistants on the male side and one on the female side, elevated into a kind of matron, was able to oppose the physician at every point; had the whole control of the attendants, and the ordering of all that was called their moral treatment. The results were instructive, and may be remembered even now with advantage. Instances of mismanagement became numerous and lamentable. Restraint was permitted to be abused. Secrecy became the rule of the asylum. An excessive economy prevailed in essential matters, mixed up with extravagance in others, calculated to produce a striking effect upon the uninformed public. When the happiness of the patients or their well-doing had ceased to be regarded as the first consideration, public entertainments were got up: not only were plays performed, but ballets were danced by the patients, and the excitements of strange musicians enlisted into the service of the asylum, to please excitable audiences. All Paris flocked, by permission, to see these exhibitions, the effects of which were of the worst description. It is just and necessary to draw a strong line of separation between all such public and noisy follies permitted in asylums at any time, and those social and cheerful assemblages of the patients of which all experience attests the advantage. This line will never be forgotten by a judicious physician, who must indeed ever grieve to see it overpassed; and if such scenes are enacted before him, can only be an unwilling and mortified spectator of them.

These exhibitions no longer disgrace Charenton. The physician is not required to act with his hands bound. He is not controlled in his proper functions, nor thwarted by his inferiors, nor compelled to take the best care he can of patients for whose cure and safety he is responsible by means of attendants chosen by those who wholly disregard his views. Yet I confess it occasioned painful emotions in my mind to see the visits of so good a physician as M. Foville necessarily almost limited to a formal tour of inspection, accompanied throughout by other officers, during which there is only time for a few words to be spoken to a certain number of the patients. In such visits the only part of the treatment which is fully attended to is that which is merely medical. The general moral treatment, based on physiology and psychology, and which is the true domain of the physician who treats mental disorders, is not wholly neglected, for even a few words or a friendly salutation, or a kind look form parts of it, and by repetition parts of important effect; but still there is much left undone that ought to be done, and would oftentimes be curative. Esquirol, to whom all that is required from and in the physician to an asylum was thoroughly and practically known, observes that "it requires a particular cast and character of mind to cultivate this branch of art fruitfully; that the physician must have much time at his disposal, and make, as it were, an abnegation of himself. . . . . He who would be useful," he adds, "to the insane, should visit them many times in the day, and even during the night; and not be satisfied by a morning visit, as in ordinary hospitals." The necessity for this cannot, of course, be always readily comprehended by the governing bodies of such institutions, who, whatever their intentions, are not always prepared by education or habits for appreciating the most delicate of tasks that can be confided to a physician. The physician alone knows what might still be attempted; what might still be achieved. The withholding from him the full privileges of doing good is not to be viewed as a matter merely affecting the dignity or pride of a doctor. Whatever limits his authority and lessens his opportunities of exercising his skill for the benefit of his patients, seriously interferes with their comfort, and has a direct tendency to retard the recovery of a great many of them. I

am convinced that the slow progress of knowledge in relation to mental disorders, and the error and uncertainty prevailing respecting the effects of all kinds of medical treatment, and the safety of disusing mechanical restraints, is more to be attributed to the want of opportunities of constant observation than to any other cause. The insane require to be very frequently seen in order to show the actual result of what is ordered; and this can only be done by medical men who live under the roof with them. For this, among many other reasons, the physician to an asylum should be resident, and enabled to pursue his own views of treatment consistently. From the moment of admission the patients should feel that their destiny is in the physician's hands. He should receive them, place them in fitting wards, see them frequently, and become their friend and their guide. All their treatment should be dictated by him and by him alone. He should have full power to protect them from all unfavorable influences, and to extend to them every aid to the renewal of those energies which are lost. They should form a family of which he is acknowledged to be the protecting head. He should know, and be understood by his patients to know, how every part of their treatment is conducted. They should see him when they are collected at their meals. He should often observe their appearance when employed. He should know how they are lodged. In all their recreations he and his family and his friends should take an occasional part. He should join them in their humble devotions at the throne of their Maker. Exclude the physician from all this, and he is deprived of some of the highest attributes of his profession. Supposing him to be capable of conceiving a perfect system of management, nothing should be permitted to interfere with it, lest it become deformed in execution and futile in result. When this is done in all asylums, and not before, it will be seen how much more can be effected for the insane than is yet accomplished in the most celebrated of such institutions. Then, and not until then, will any one of them present a perfect example of an asylum in which the influence of the intellect and the feelings has superseded that of mere physical power.

On the day after my visit to Charenton, I had the gratification, by means of M. Foville's introduction, of an interview with M. Ferrus, whose name has been so long and honorably associated with institutions for the insane; first, as physician to the Bicêtre, and subsequently as inspector-general of the asylums of France. The immense ameliorations which have been made or are in progress in the French asylums are understood to have received a great share of their impulse from the zeal and ability of M. Ferrus. He possesses an extensive and exact acquaintance with everything connected with their administration, conjoined with that practical knowledge of the treatment and general management of lunatics which best directs the judgment even of the most intelligent and humane public functionaries. Conscious as M. Ferrus cannot but be of the extensive reforms which he has promoted throughout the asylums of France, he appears wholly free from the weakness of believing that every desirable end is yet accomplished; and he listened with a very gratifying appearance of interest to my account of what had been attempted at Hanwell and in other English asylums, and expressed, with a sincerity that could not be doubted, the pleasure he derived from hearing that we had endeavoured to take one step more in improvement by the abolition of all physical restraints. M. Ferrus is the president of the Académie de Médecine, an academy so liberal in its construction and so influential in its proceedings, that it is a just matter of surprise as well as of regret that we have no analogous institution in England. At one of their Monday meetings, I had the pleasure to witness the assemblage of about 300 members and visitors, comprehending some or most of the distinguished physicians and surgeons of Paris; and of hearing some of them take an animated part in the discussion of the claims of M. Guerin to the merit of practising a new and successful operation in the treatment of distortion of the spine. On this occasion, also, I had the great pleasure of once more seeing M. Pariset, whom I first knew when he was physician to the Salpêtrière, now a veteran among physicians who have devoted themselves to the insane, and enjoying a well-earned fame. His age is now considerable; but he is still one of the secretaries of the Academy, and his characteristic activity has not wholly deserted him; and when proper occasions animate him, as in the *Eloge* of M. Esquirol, lately read before the Academy, his wonted eloquence is still shown to remain. I introduce his name, when speaking of the asylums of his country, with deep respect. Throughout a long professional life he has been distinguished by a devotion to scientific inquiries, which has made him regardless of danger and fatigue amidst pestilence and death; and in his treatment of the insane, his views have been invariably marked by warm philanthropy, tempered with soundness of judgement. If he has been gifted with the liveliest powers of expressing and enforcing his opinions, he has on every occasion devoted those powers to the advocacy of humanity.

### ROUEN.

### ASYLUM OF ST. YON.

Before M. Foville's removal to Paris, he was already distinguished as the physician to the Asylum of St. Yon, at Rouen, to which office he was appointed on the recommendation of Esquirol, who has commemorated the order and discipline which he there introduced. By M. Foville's advice, I was induced to quit Paris for two days, for the sake of visiting an establishment, the general plan of which is understood to be conformable to the views of such buildings entertained by M. Esquirol himself; and which also was one of the first in which those reformations commenced, to advance which he so greatly laboured.

The railway makes a journey to Rouen from Paris, and back, easily performed in a few hours; and to those remembering the journey from one place to the other as the wearisome occupation of a whole day, the improvement thus effected conveys almost a miraculous impression. It requires some effort to allude to this short journey without remarks unconnected with the object of my letter; so interesting and so beautiful are those scenes of Norman history and romance through which the traveller is rapidly transported, as in a dream. The flight and traces of time, too, are never more strongly brought to the traveller's mind, than on revisiting any populous town after many years. When I saw Rouen more than a quarter of a century ago, all was ancient in that singular town, and a frail bridge of boats alone connected the two sides of the broad river that runs through it. Thirty years of peace have given rise to new edifices: two fine bridges of stone cross the Seine, and a railway and its accompaniments enliven the southern suburbs, formerly so dull and deserted. When I then first saw the magnificent cathedral, it was in the blaze of an autumnal day, on a Sunday, during the morning service; and the Cardinal Cambaceres sate on his throne, in scarlet robes, in appearance not unlike the portraits of Wolsey: the sunshine streamed down on a large congregation, surrounded by all the pomp of Roman worship, and the vast aisles were filled with the sacred melody of organ and choir. It happened that *this* time I visited it quite at the close of a dull November day, in fog and rain, when there was scarcely light sufficient to find the way up to the grand altar. Here and there a single candle threw its beams into the sublime darkness of the building, and a few persons might be seen kneeling here and there in solitary and tranquil devotion. A few figures now and then passed across the gloom; priests and their attendants, or devotees coming to evening prayer. The lightest step was audible; and in this still obscurity I walked reverently round the sacred edifice, and came to the tomb wherein now lies that same Cardinal Cambaceres "in dull, cold marble;" and, thus reminded of the swift passing of years and of men, could not long forget that the allotted time for performing any task proposed to be accomplished was narrowing too, and must not be wasted.

The Asylum of St. Yon, an ancient manor house, originally without the boundaries of the town, and at a subsequent period the property of one of the religious orders, and used for the various purposes of religious instruction, the education of indocile and troublesome young persons, and also for the reception of epileptic and insane people, but converted at the time of the revolution into a prison, finally became an asylum for the insane of the department of la Seine Inférieure about twenty years since. The plans of the new buildings rendered necessary were submitted to the inspection and opinions of M. Esquirol, and of M. Desportes, who at that time held the office now filled by M. Battelle. It is approached by a long street in the southern quarter of Rouen. The gate of the asylum opens from the street, and the front of the church is next to it, being at once the church of the parish and of the asylum. The asylum, and the gardens and cultivated grounds are extensive, conveniently subdivided, occupying a large triangular space within high walls, and not much overlooked by surrounding houses. It is not very easy to form an accurate notion of the disposition of asylums arranged like those I have lately seen; in which, instead of the intelligible simplicity of a single mass of buildings, there are several and variously disposed edifices or quadrangles, or separate houses. But at Rouen I had the assistance of a large plan, kindly given to me by the director, M. Boutteville, in the absence of M. Parchappe, the physician to the asylum, an absence which I very much regretted.

On entering the asylum, a considerable building is first approached, forming a square, with a garden in the centre; this building is partly occupied by the sisters of a religious order, who in this asylum perform all the offices of nurses or attendants for the female patients; and it is partly devoted to the two infirmaries, and to day-rooms and dormitories for quiet patients. It also contains the bureau; and in the basement there are store-rooms, workshops, kitchens, and various offices. This building appears to be a part of the older constructions, and not in exact accordance with the design of the more recent parts of the asylum. To the right of it, and separated by cultivated grounds, are three quadrilateral inclosures, each distinct from the rest. These contain the female patients; two of them being for the moderately tranquil, or those described as under treatment; and one, the most remote, for the refractory or violent. There is a separate building for such of the female patients as are dirty. On the left of the central building, similarly separated from it, are two of the same kind of quadrilateral inclosures, for the male patients; one for the moderately tranquil, the other for the more agitated; and there are small separate wards at the outer extremity of one side of each of the quadrangles, and extending beyond it; one for the dirty, and one for the extremely violent; and to each of these there is a separate airing-court. On each side of the asylum there are separate buildings, with pleasant gardens, for the patients who pay for their admission, maintenance, and treatment. The laundry and baths form also detached buildings; and to the baths there are separate covered galleries or corridors, but from the two sides of the central building only. Every court, and the interior of every quadrilateral inclosure, is planted as a garden, so as to form an agreeable walk. All the inclosures have buildings on three of their sides only, the fourth being bounded by a high palisade, in all cases affording a view of the outer garden, or farm-grounds; and the buildings on the three sides consist only of one floor. The cells or sleeping-rooms of the patients have large windows towards a colonnade or open corridor, which is continued on every side of the quadrangle, and the doors of the rooms are opposite to the windows, and open into a covered and closed gallery, lighted by high windows.

An asylum thus arranged affords apparent facilities for the subdivision and classification of the patients; the accomplishment of which is, however, often impeded, always limited by numerous considerations, only intimately known to those who live in asylums; but, above all, by the size of the dormitories in an asylum, which, if large, renders a proper classification of the patients quite impossible. When the patients are all on one floor, and have no stairs to climb and descend, many of them may be more easily induced to take exercise, or can more easily walk out, or be carried into the open air. The attendants are also nearer to each other, and may support each other more promptly in case of accidents or sudden danger. Windows are not so far from the ground: but the inconvenience of having so many detached edifices, not even connected with covered walks, and some of them necessarily remote, and all separated from the central or chief house, from the residences of the officers, from the surgery, the store-rooms, the baths, the kitchen, the laundry, and from the chapel, are also obvious. The general inspection of such an asylum must be very difficult; and the sudden emergencies of insanity and sickness can scarcely be efficiently met. The eye of the superintendent must often be wanting, and the patients left to the unchecked control of attendants. If the windows are less obnoxious to accidents, they are also more favorable to escapes. But the greater expense alone of such erections will probably always preclude their general adoption.

There are more than 600 patients in the Asylum of St. Yon: a certain number of them are received for annual payments, varying from 450 to 1500 francs (from about 18*l.* to 60*l.* English). The rest are patients belonging to the department of the Seine Inférieure, or to the departments adjoining; for each of whom the departments pay respectively 15*l.* and 18*l.* per annum.

The general management of the asylum appears to be very good: much attention is paid to the employment of the patients; some to their general instruction also; and some to their amusement. The male patients are chiefly occupied in the gardens and grounds, where I saw many of them very busily and cheerfully engaged in making various alterations in progress at the time of my visit. A small weekly allowance of money is made to those who work, which constitutes a little fund to aid them when they leave the asylum, on recovery. The gardens and promenades are kept in admirable order by their industry. The female patients are also very industrious. In one room there were forty engaged in needlework; and there were several in other rooms. Some of the sisters were in each of these apartments, sitting with the patients, and directing them in their occupations: the composure and mildness of manner of these excellent persons towards those to whose care they devote themselves, were answerable to the general character they have obtained as nurses or attendants on the insane. Several of them are, it may be supposed, superior in station and acquirements to the ordinary nurses, and no doubt they are also treated with proper consideration, without which no good nurses can be retained in any establishment.

The same extreme tranquillity, which I have already noticed as surprising me at the Salpêtrière, was observable in the wards of the St. Yon. I imagine that this great degree of quiet and silence in some of the French asylums, and which I have also remarked in some English asylums, although certainly not at Hanwell, is not altogether a proof of excellence; but arises from the patients not being habituated to seeing many visitors, or to being addressed and noticed, or allowed much of the freedom of conversation, or discourse rather, in which so many lunatics take delight; and which certainly calls for occasional restriction. My partiality for Hanwell perhaps influences me in my preference of what I witness there to the forced decorum and reserve to which I am now alluding. I certainly do not object to seeing the officers, who enter the wards, surrounded by patients eager to communicate their joys or their sorrows, to prefer their requests, or often just complaints, and to make their mindful and affectionate inquiries. These opportunities form the principal relief of the terrible monotony of years passed by those confined to one building and its grounds, many of them for life. The mere expectation of such visits constitutes much of their happiness, and whatever interferes with them is a denial of comfort to the patients in their captivity and their affliction. Nor can I consider the discipline or the prosperity of an asylum endangered if, on entering the workrooms, the knitter or embroideress suspends her occupation, or the tailor leaves his shopboard, or the carpenter desists a few moments from hammering and planing, to exchange a few cheerful words with a visitor; whereas, to walk through workrooms and wards full of insane persons and see no head raised, and find all silent, appears to be somewhat unnatural, and makes one suspect that many feelings are forcibly repressed which to express would be a pleasure or a relief that ought not to be withheld. The visit of the chief officer of an asylum ought to be the chief medicine; it should be the principal among remedies; the daily repetition of it, his manner, his words, his demeanour, his voice, are of more influence, for good or evil, than all the stores of the pharmacopœia. To ensure good effects demands a great devotion of time, considerable alacrity of mind, and a genuine and never failing compassion; none of which qualities are called for to walk through a silent crowd of toiling people. And if the officers habitually pass the patients by in silence, with small recognition or regard, or merely order medicines, baths, or means of repression, it is not to be supposed that the attendants will be habitually considerate and kind to those scarcely deemed worthy of the notice of the officers to whom they are responsibl e.

These latter observations are not entirely without application to the asylum at Rouen; for although the well-arranged and able reports of M. Parchappe, the chief physician, show that he entertains both humane and just views concerning the influence of recreation in the treatment of insanity, encourages their instruction in reading and singing, and believes that, with certain prudent limitations, music and other entertainments become remedial, and, in short, appreciates all the best principles of moral treatment, there is still visible at St. Yon an adherence to a method of discipline which savours of the austerity of an ancient religious house. In the regulations, whilst nothing is said of preventing the beginning of strife among the patients, it is ordained that if quarrels arise they are to be repressed with severity and reprimands. Baths are enumerated among the means of repression, which means punishment, and not among the resources of treatment. The douche is only employed as a punishment. No various dress, no decoration, no eccentricity of appearance, however harmless, is allowed; and at the visit of the physician, who should surely come among his patients as a father among his children, it seems that the patients are arranged in ranks, the men standing up, the women seated, but all expressly forbidden to make complaints. A profound silence is prescribed until the physician's visit is at an end. It is needless to say that these regulations must keep the physician a stranger to the character of many under his charge, and if promotive of discipline and submission, are totally exclusive of friendly intercourse, and the kind of consolation so often required at the physician's hands. If we were to suppose such regulations introduced at Hanwell they would give to that asylum, compared with its present appearance, the aspect of a mausoleum for the dead.

The employment of the patients of St. Yon has received careful attention. Needlework, straw-hat making, stocking-knitting, and the business of the laundry, seem chiefly to occupy the female patients; and the men are not only engaged, as has been already mentioned, in the grounds, but also assist the cooks, the butcher, and in the general cleaning and service of the wards. The patients dine together in the different day-rooms, and are allowed the use of ordinary knives and forks. They sleep in dormitories, containing each about thirty beds, some attendants sleeping in each dormitory. All these rooms were particularly neat and clean, and the beds excellent and in the best order. The ventilation through old fashioned windows, and the warming of the asylum, appeared defective. The fire-guards are of the ancient kind, made of strong iron bars; but I saw no iron fetters hung upon them, such as I lately observed with surprise in the asylum at Manchester. At the Rouen asylum, the opinion of dormitories having a calming effect on the patients is strongly entertained, an opinion in support of which our experience at Hanwell certainly affords nothing, the effect of dormitories seeming to be always quite the contrary.

Although the general appearance of the patients whom I saw was comfortable, and they were most of them well clothed, I think I caught glimpses in the quadrangles on the female side of the house, which I was rather rapidly led through, of quiet-looking patients in the camisole or strait-waistcoat; and in one noisy room there were several so dressed. It is stated, indeed, in one of M. Parchappe's reports, that the strait-waistcoat is seldom used on the male side of the house, but that altogether 3 in 100 are habitually so restrained in the asylum. The same plan would make it necessary to introduce thirty strait-waistooats into the Hanwell asylum for ordinary and daily use. Neither the management of patients without restraint, nor our unobjectionable method of secluding patients for a time in their usual sleeping rooms, or in a padded room, according to circumstances, seem yet to be understood. But seclusion is actually practised in a certain manner, resembling what I remember in the old asylums of England less than 20 years since; the patient secluded being left entirely naked, and lying, if he chose it, in his deep crib of straw, covered over with it for warmth. This is at least less objectionable than restraint, although a patient so left is seldom properly attended to, but is permitted to lie as untended as an animal in a straw-yard. Washing, if thought of at all in such circumstances, is rudely performed, and perfect cleanliness is out of the question. The sympathies of the attendants are not strongly excited in such cases, and when once the crib and the straw are resorted to, their convenience prolongs the period for which the patient is consigned to them. With a patient in simple seclusion, liberation is easily effected: the door is opened, and he walks out. A patient who has wallowed naked in straw cannot be produced without much more trouble, and the trouble is deferred. We entered the room of a man thus situated at the Rouen asylum; he talked quietly, and made no attempt to get out of his crib; but he was evidently in an excitable state, for which simple seclusion would have been an appropriate remedy. But when the door was shut upon him, and fastened by two large noisy bolts, the question naturally presented itself—how long will this man remain there?

I should hesitate to offer such a question; but if I had up to that time wanted much experience in the neglects incidental to the restraint system, I could not have been insensible to the danger existing in asylums of becoming an indifferent spectator of restraints too long continued, when, in this same asylum of Rouen, where every kind sentiment is expressed, and only entire non-restraint condemned, I saw a man in another cell lying in a straw crib, having a strait-waistcoat on, so as to secure his arms and body, and his feet bound to the crib, who had lain so dressed, so secured and bound, *nine years.* He doubtless, lies there still; he will lie there until death unbinds him. I had read of such things, and I had seen in our own country gross cases of prolonged and cruel restraint; but I felt humiliated and useless as I turned away and left this man, an old mariner, moving his head and body for ever a little to one side, and a little to the other; and uttering a few incoherent words, but distinctly, so as to leave no hope of his being quite unconscious of his perpetual bondage.

I acquit the director and physician of the Rouen asylum of all intentional or conscious cruelty in their management of this case. They are firmly convinced that such treatment is absolutely indispensable. The director spoke most kindly of and to the poor man. So have I formerly witnessed humane English physicians, full of the same convictions, daily contemplating helpless insane patients bound hand and foot, and neck, and waist, in illness, in pain, and in the agonies of death, equally without one single touch of compunction, or the slightest approach to a feeling of acting either cruelly or unwisely. They thought it impossible to manage insane people in any other way.

This very case is quoted in a review of the Hanwell Reports, by M. Brierre de Boismont, in no unfriendly spirit, to prove that our system at Hanwell is one of philanthropic impracticability. The man is described as being disposed to suicide: he would beat his head against the wall; he would roll about the floor; he would tear his clothes. These are excuses for restraint which, without one exception, experience at Hanwell has taught us to be trifling. A padded room; a dress of materials not easily torn; frequent refreshment in the open air; exercise; kind words and good food, will not only prevent immediate danger in such cases, but eventually remove the cause of danger. Restraint is but half a remedy. Granting that it excludes immediate danger of suicide (which it does *not*,) it does nothing for its cure; nothing to alter the suicidal condition of the mind. It must therefore be prolonged as long as the patient lives.

Various other cases are quoted by recent French critics, in no illiberal spirit, and without any attempt at severe criticism, to show the folly or risk of abolishing restraints. In one case, the physician resolved, however, to try. A suicidal gentleman was left in bed, unbound, but watched by two attendants, who had nothing to do but to watch him. In their watchful presence, it seems, he tore his shirt and strangled himself, without their perceiving it until he was dead. The physician looked in at the door of the patient's room, to make inquiries; and the attendants reported all quiet and comfortable: "he slept well;" and it was at length found that he slept the sleep of death. But surely the attendants in this case must have been scarcely more awake. They were men accustomed, perhaps, to rely on restraints; and unaccustomed to watch. Attendants of this kind in London have been known to tie up patients in their own dwelling-houses, in private families, when the physician has gone away; and then to go to supper, or spend the tedious hours of the night at cards. The patient becomes exasperated, and the cords are tightened. He continues restless, but he cannot get up. In speaking of such things even from an actual knowledge of the facts, I feel that I shall be suspected of exaggeration: I am now simply transcribing from the history of the case of a gentleman who afterwards recovered in a public asylum, to which he came lamed with restraint, and in a state of great irritation. With such attendants, to dispense with restraints is impossible.

That it is in no other sense impracticable may I think be asserted without the charge of presumption, now that for more than five years no hand or foot has been bound in the Hanwell Asylum by night or by day ; a period during which more than 900 admissions have taken place, including patients exhibiting all the varieties of insanity, and all degrees of violence, and all imaginable diversities of difficulty. It is our rule, however tightly tied up the patient is brought to us, to liberate him at once; and the experience of the period I have mentioned proves, beyond the possibility of doubt, that the rule is safe as well as humane. Determination in the director, and willing obedience in the attendants overcome every obstacle. In a former part of these observations I mentioned with satisfaction the fact that one of the medical officers had lately gone from Hanwell to take charge of the lunatics in Ceylon. Amidst difficulties inconceivable in Europe, this excellent physician (Dr. James George Davey,) has resolutely commenced acting on the principles which he has so long seen in salutary operation ; and the results promise to be equally satisfactory. One of the first cases which attracted his attention was that of a male patient sitting on the ground in a sort of cell or sty, with one of his legs fastened into a hole in the stocks: he was uttering horrid execrations ; excited, frantic in the highest degree. His eyes were glassy and white, his countenance depressed and sunken; and his bodily powers almost exhausted by unceasing efforts to get loose. Here was a seeming subject for years of thraldom. Dr. Davey immediately caused the door to be opened, went up to the poor maniac, and after a little time succeeded in gaining his attention. His unexpected kindness was so alleviating in its effects, that in a few minutes the patient, liberated from the stocks, was walking up and down the court with his kind liberator. Some refreshment was ordered for him, and Dr. Davey left him after a little time, to use his own expressions, "as quiet and peaceable as an infant." Such are the effects of courage and of kindness; and the time will soon, I trust, arrive when their operation will penetrate into all cells, wherever existing, in which the victims of years now lie in hopeless restraints, like the poor man at St. Yon.

### PRIVATE ASYLUMS OF PARIS.

Before leaving Paris I was obligingly allowed an opportunity of seeing three of the principal private establishments for insane persons ; one of them being superintended by Drs. Falret and Voisin, one by Dr. Leuret, and one by Dr. Mitivié. The arrangements in these establishments, and the manner in which they are conducted, are conformable to the high character of these distinguished physicians. In each there are several buildings, facilitating the division of the male and female patients, with the necessary subdivisions of the tranquil, the noisy, &c. ; and in each a chief or central house of residence for the medical superintendents, which is also a place of re-union in the evening, or at other times, for such patients as can appreciate or derive benefit from social advantages. Each of these establishments possesses grounds of considerable extent, agreeably laid out; and the appearance of the patients and their attendants, always one of the most satisfactory tests of the real management of an asylum, was in each such as to afford every assurance of their enjoying all the comforts compatible with their malady.

The large establishment of MM. Falret and Voisin, at Vanves, about three miles south-west of Paris, has numerous recommendations in its site, its extent, and its disposition. I may describe it in general terms as a park, containing eighty French acres, and comprising a great variety of ground and pleasing combinations of scenery ; whilst on the borders of this park there are several houses, which constitute the residences of the patients ; almost every house that overlooks the grounds forming a part of the asylum. Some of these houses are so detached as to enable the proprietors in certain cases to receive the families or relatives of the patients as temporary residents. The various buildings also enable them to offer distinct residences to some of their patients, who can enjoy all the comfort of living in a house which seems their own, and have yet the advantage of a salutary superintendence. I was conducted through all these buildings, and over the park, by M. Voisin, accompanied by M. Battelle and M. Mallon. We saw, I believe, nearly every patient, and had an opportunity of estimating the general advantages of the establishment by afterward meeting some of the patients at dinner, with the families of the two physicians, and M. Christophe, one of the chaplains of the Salpêtrière; and of passing the evening with them. M. Falret has visited England, and is well known to many English practitioners. He is a man of fine and commanding appearance, sedate in his general demeanour, but cheerful, and in conversation earnest and animated. His various published works are of the highest character. Not to mention others, of greater extent, the article *Maladies Mentales* in the 'Dictionnaire de Méd. Usuelle,' contains every good principle of moral treatment, expressed with a justness and force that show M. Falret to be a worthy follower of M. Esquirol, his preceptor. M. Voisin is contrasted with his colleague by an ardour and vivacity conspicuous in his writings and his discourse, and remarkable even in his own country; his feelings are acute, his perceptions rapid, and his expressions forcible and even enthusiastic. One might almost say that these two benevolent persons have been providentially united in their undertakings for benevolent purposes; and they have been thus associated, amicably and usefully, more than twenty years. M. Voisin is sensibly alive to the important functions of a physician who undertakes to cure or alleviate infirmities of the mind; he speaks on this subject with an energy and fluency by which the hearer is carried away; but at the same time with reason and good sense, happily in him combined with a zealous and rare humanity. It is, especially, impossible to listen unmoved to his eloquent appeals in favour of the idiotic class of patients, for whom he has done and could still do so much. His views receive, perhaps, a beneficial modification from the composed and reflecting habits of M. Falret; but in philanthropy both seem to be equal. The mere eulogy of good men is grateful in itself; but the permanent pleasure derived from intercourse with physicians of this character, devoted to the care and cure of the insane, arises from the conviction that under their auspices every measure will in time be fostered and established by which the miseries of insanity can be lessened.

In the course of our inspection of this asylum, we met some of the patients in the grounds; in the pleasant shade of shrubberies, or by the side of a clear and refreshing stream which runs through the estate, or on the various heights, which afford various views of it, and of the neighbourhood. We also passed through several rooms of some of the houses at the dinner hour, and found various parties of the patients sitting at that meal, with every comfort belonging to their station. We were shown a great number of apartments, sitting-rooms, and bed-rooms, all neat and well furnished, most of them inhabited by patients, several of whom, by their readiness to converse, reminded me of patients with whom I am more familiar at home. In a grove, in a beautiful part of the park, there is a small and elegant chapel; and in another part of the grounds there are farm-buildings. Gardens full of flowering shrubs, and refreshed by fountains, divide and screen some of the houses from the central park; and the objects seen from the windows are of a nature to please and soothe those whose sphere of active enjoyment is the most limited. Diversified means of mental recreation seem also to be provided, and occasional reunions in the larger drawing-rooms of the central house present objects of pleasant anticipation, and are productive of more than transient benefit.

But still, MM. Falret and Voisin will, I hope, forgive me if I add, that I deeply lamented to find the degrading evil of physical restraint still permitted, although only in a few instances, to spoil the beautiful design of their asylum. That such enlightened and benevolent physicians, living in a house more than usually full of resources against all the violences and caprices of madness, should still avail themselves of this, the lowest, the coarsest, and the worst, does but prove the force of habit in resisting the progress of improvement. The spectacle of patients bound up in compressing dresses, painful to the body, and painful to every mind, as long as any mind is left, appeared even unnatural in an establishment presenting, with this exception, a kind of model of an asylum for the higher classes of patients. M. Voisin himself seemed, I thought, quite in an unnatural position when he was unbinding, and untwisting, and refixing, and demonstrating the miserable complications of linen, leather, and iron, on a poor lunatic's body, with a view of controlling and healing the rude outbreaks of the feelings, or the wild delusions of a disordered and afflicted mind. I feel, however, a comfortable assurance that the excellent directors of the asylum at Vanves will soon have banished what now appears to be only an inexplicable anomaly in their great plan, and at variance with every other indication of their own feelings and convictions.

M. Leuret's asylum is situated in the south-western suburb of Paris, on the south bank of the Seine, the grounds being bordered on the west by the large space called the Champ de Mars. On the high bank of the river, opposite to it, are the houses and gardens of Chaillot and Passy, the most agreeable of the suburban villages of the French capital. The opinions with which M. Leuret's name is particularly associated in England, scarcely do justice to his humane and sensible character. For a time, at least, the idea of him was that of a physician who undertook to expel all delusions by the force, not of argument, but of a column of cold water directed to the outside of the victims's head. I believe he does not now very sedulously follow the rigid rules once seemingly advocated in his writings respecting the insane; but finds that their infirmities do really sometimes demand indulgence, and that some of their delusions actually resist the douche. It is difficult to account for the advocacy of so kind a physician, for such he really is, of a system characterized by no less an authority than that of M. Pariset, conjoined with M. Esquirol, in a Report presented to the Academy of Medicine, as one of intimidation, mixed with mockeries, reproaches, and physical pains, followed, indeed, by diligent and kind attentions, with the view of compelling patients to disavow or resign their morbid ideas. Like all other strange modes of practice, this was probably not destitute of some portion of rational foundation; but in its general character there was an evident and dangerous resemblance to cruelty; and its general success would have been truly unfortunate, tending, no doubt, to bring back the ancient method of beating lunatics into their senses, and flogging them into calmness, especially vaunted once in Scotland, and practised everywhere else.

On two occasions on which I visited M. Leuret's establishment, every opportunity being given to me of seeing all parts of it, I was gratified with the appearance of perfect order and comfort everywhere visible. It consists of an excellent house, which is separated from the street by a spacious, paved court-yard. Behind the house are several acres of garden and pleasure-ground, in which I was glad to see that M. Leuret had succeeded in persuading some of the patients under his care to occupy themselves; for the difficulty of doing this makes the general indolence and listlessness of private asylums very oppressive. On one side of the grounds there is a detached house for female patients, with provision for their proper classification; and on the other side of the grounds there is a house for male patients, with some rooms and a small inclosed airing-court, especially designed for the noisiest of the patients, or for those incapable of enjoying the pleasure of being more at large in the grounds or gardens. I understood that it was M. Leuret's custom every evening, or at least frequently, to invite a certain number of his patients to the central house; in the handsome apartments of which they assemble, music and other recreations being provided for them. The attention evidently paid to the state of the gardens, and to the walks for the patients, and the cheerful appearance of the buildings, and of the whole establishment, afforded strong evidence of the general efficiency of the superintendence of this asylum, and would exclude almost every unpleasant association from the mind of a visitor. I had, of course, no opportunity of becoming acquainted with the details of its management; but the appearance of all the patients whom I saw was highly satisfactory.

M. Mitivié's kind invitation afforded me a much-desired opportunity of seeing the private establishment at Ivry, which was that of M. Esquirol, and where, consequently, everything speaks of that illustrious physician. Ivry is a village a few miles east of Paris, south of the Seine; and M. Mitivié's asylum is in a retired but agreeable situation, not commanding extensive views, but wholly surrounded by rural objects. There is here, as in the other private asylums of Paris which I visited, a central or chief residence, with detached residences for the different sexes, and for patients variously affected. But at Ivry some parts of the establishment present M. Esquirol's favourite form of quadrangles, or rather of quadrilateral inclosures, the buildings of which, of one story only, are placed on three sides, the fourth side being bounded by a high iron palisade, through which the patients can see the lawn, the gardens, or the fields. The interior of these inclosures is sown with grass, and there is a broad covered walk on every side. It is scarcely necessary to add that all the arrangements of this asylum are carefully adapted to the class of patients received. The whole establishment has an air of calmness and repose likely to be grateful and beneficial to the patients sent thither, as to a retreat from the ever-agitated capital to which it is so near. M. Mitivié is a man of quiet and somewhat reserved manners, but extremely well-informed, judicious, and candid. He is the nephew of Esquirol, and it was a singular pleasure to me to hear from him some traits of the character and habits of his illustrious uncle, with whose writings I have for many years kept up an almost daily acquaintance. All that I heard answered to my previous conceptions of the simple philanthropy, sagacity, and industry of that great follower of Pinel. His published works exhibit, I believe, but a small part of the labours of more than thirty years concentrated almost upon one great object; and to which the large collection of crania and casts at Ivry, as well as numerous drawings illustrative of the characters of insanity, and a vast collection of plans of asylums attest the constant activity of his attention. I had passed a whole morning in seeking, in all the print-shops of the Quai Voltaire, a good print of Esquirol, before I found that the only good one, from a painting by Pichon, was the private property of M. Mitivié; and I consider myself much enriched by being by his kindness made the possessor of a copy, the interest of this present being increased, if possible, by my receiving it in the house in Paris which was the residence of Esquirol, and is now inhabited by M. Mitivié; and in the library where Esquirol wrote, and by the chair in which he usually sat, and in which he was painted. In this portrait, Esquirol is represented as sitting by the bust of Pinel; and both the bust and portrait are in M. Mitivié's house. The medical men of other countries have sometimes reproached the French physicians with a contented ignorance of the writings of all medical authors out of their own country; but in M. Esquirol's well-arranged library, and which still seems as if it were his, I was gratified to see nearly every English work of standard reputation, not only on insanity, but on all other branches of medical science. The collection of works on insanity appeared to be most complete. The house is in a quiet street in that silent quarter of Paris near the Garden of Plants, where so many scholars, naturalists, men of science, artists, and other studious persons most congregate; in which part of Paris Cuvier passed so many years devoted to immortal labours, and where so many great men have spent their lives in retired devotion to the highest pursuits that can engage the human mind. The windows of what was the study of Esquirol open on a small and pleasant garden; and in this room, after the numerous and active duties of the day, he used to occupy himself in writing at night; a circumstance to which M. Mitivié ascribes some alleged defects in his style, of which a foreigner is, I think, scarcely conscious; the descriptions in his pages being generally not only so characterized by his penetration and wisdom, but written so clearly and with so much spirit, that there are no medical works which the reader peruses with less fatigue. In his eloquent commentaries, the diligent student finds at each reperusal fresh proofs of his admirable powers of observation, and of his sedate and excellent judgment; and those who are most constantly with the insane, and most occupied in devising means of promoting their comfort and cure, can best appreciate the innumerable proofs his works afford of an active mind, animated in the pursuit of knowledge of the wants of the insane by a benevolent heart. His publications, his journeys of inspection, his unwearied literary efforts, carried the first reforms into all the provincial asylums of France. His lectures and his writings next diffused sound principles throughout Europe and throughout the world; his pupils were selected to superintend the asylums of the countries from which they had resorted to Paris to learn under so great a master;* and his views still evidently animate those of his countrymen who now enjoy opportunities of acting upon them. He appears, like his great predecessor, to have been often harassed with opposition, and to have had the mortification to see his opinions for a time disregarded or overruled; but for both, death, the great benefactor and ultimate repairer of human wrong, has finally "extinguished envy, and opened the gate to good fame;" and I believe all the living physicians of Europe, who hope to be remembered among those who have benefited the insane, gratefully acknowledge that their best efforts have been stimulated, if not altogether suggested, by the examples of Esquirol and of Pinel.

There are other private asylums in Paris which I should have been glad to visit; but my time was too limited to permit me to do so, or even to pay my respects to many of the Parisian physicians whose names adorn our profession.

Upon the whole, I prefer the aspect of the French private asylums to those of England, with a few distinguished exceptions. I think the arrangements made for the reception and treatment of private patients are more systematic and better than in our ordinary private asylums. Those, however, which I have described, are to be considered among the best. I believe it would much conduce to the comfort of patients of the richer classes if all private asylums were so regulated or so enlarged as to give them a more special adaptation to their object, and the advantages which are now only to be found in public asylums where either the patients are received gratuitously or for very moderate payments. The attractive idea of giving an entirely domestic character to a private asylum is either delusive, or only compatible with a plan which excludes all troublesome patients. Without this exclusion, the proprietors of the asylums and their families, and all their most reasonable patients must be subjected to constant annoyances, by which no one is benefited; for it is the loss of the power of conforming to ordinary customs and of enjoying or contributing to social comfort, that is the actual cause of the patients being sent to the asylum from their families. Very few of them become fit for continual and intimate association until they are decidedly convalescent. Those who have not been marked by any natural feebleness of mind may gradually be restored to these social capacities; and perfect restoration to them constitutes their cure: but to endeavour to force such restoration is useless. The chief error of many of our English private asylums, partly arising from defective views in this particular, is that their general construction is unsuited to the purposes to which they are applied. There may be half a dozen exceptions to this remark; where the proprietors of such houses are wealthy. More commonly we find large dilapidated mansions converted into asylums; badly situated and much out of repair. An inadequate establishment is meagerly stretched out to fit the great mansion, and the exterior signs of decay prepare the visitor for the discomfort and gloom within, which is too well calculated to take all heart out of the arriving patient. Cold rooms, tawdry furniture, dirty passages, and various other peculiarities seem, even during a temporary visit, to shut out the hopeful world, and give prevalence to dismal thoughts alone. All these incongruities arise from the attempt to do what cannot be done, and to avoid what would alone be advantageous, assimilating the house to a public asylum. As it is, the patients consider themselves mocked by the semblance of a private house, and lose the advantage which arises from a conviction that they really are not considered to be of perfectly sound mind.

* Discours prononcé sur la Tombe de M. Esquirol, le 14 Decembre, 1840, par M. Falret, son ancien élève; Paris, 1841.

This error is, however, venial; and seems to have sprung from a laudable wish to deprive a private asylum of every repulsive feature.

Far worse evils exist in private asylums, for which at present there appears to be no remedy. I am convinced, from information which reaches me through many channels in the course of my public duty at Hanwell, that there is no gross neglect, no wanton cruelty, which is not still practised in some of these institutions. Every kind of vexatious severity, every exasperating meanness, may be employed to torment the inmates of a private asylum; their food may be bad; their attendants brutal; they may be doomed to the dullest monotony, stinted in fire and candle, and mental occupation or relaxation, or proper bodily exercise, exposed to insufferable insolence from servants, or even neglected as to cleanliness; and yet the low cunning of those who trade in insanity may continue to make a decent appearance before the Commissioners. From what I have myself seen I presume there is no degree of coarseness, ignorance, or vulgarity which is understood to disqualify any man or any woman for obtaining a license to take care of insane persons. And over those who have a license, the Commissioners can exercise but small control. They cannot know much of the diet or the general treatment in such houses; they have no means of finding out whether in any given case an attendant has been chosen for any better quality than that of being content with low wages. Even the returns of the deaths of patients seems to be very carelessly made to them; and deaths sometimes occur in suspicious circumstances which the Commissioners should have an immediate opportunity of investigating. In public asylums these matters are better looked after; but it would be well, even in those institutions, if the minutes of the governing bodies, the list of casualties, and the various report books were submitted to official inspection. Irresponsibility is a dangerous element in the government of asylums for lunatics; and my opinion, formed and expressed long ago, has been confirmed by the subsequent experience of many years, that every insane person should in some way or other become the public care, and be looked after by responsible officers of the state, so as to ensure the hope of recovery and liberty to every curable case, and proper protection to all.

In thus giving you an account of my recent visits to some of the French asylums, I have naturally been led to comment on several matters with relation to those of our own country; and although I acknowledge the imperfection of what I have merely offered to you as sketches, I believe that some of the subjects on which I have made these desultory observations are really at this time deserving of the attention of the governing bodies and officers of asylums, and of all who take an interest in a class of persons labouring under one of the severest of human afflictions.

I remain, my dear Sir, always, with much regard,
Very faithfully yours,
John Conolly.

Lawn House, Hanwell; *Dec.* 1844.

---

